# 5-lipoxygenase mediates docosahexaenoyl ethanolamide and N-arachidonoyl-L-alanine-induced reactive oxygen species production and inhibition of proliferation of head and neck squamous cell carcinoma cells

**DOI:** 10.1186/s12885-016-2499-3

**Published:** 2016-07-13

**Authors:** Seok-Woo Park, J. Hun Hah, Sang-Mi Oh, Woo-Jin Jeong, Myung-Whun Sung

**Affiliations:** Cancer Research Institute, Seoul National University College of Medicine, Seoul, South Korea; Department of Otorhinolaryngology-Head and Neck Surgery, Seoul National University Hospital, Seoul, South Korea; Clinical Research Institute, Seoul National University Hospital, Seoul, South Korea; Department of Otorhinolaryngology, Seoul National University Bundang Hospital, Seoul, South Korea; Sensory Organ Research Institute, Seoul National University Medical Research Center, Seoul National University Hospital, Seoul, South Korea

**Keywords:** Endocannabinoid, DHEA, NALA, 5-lipoxygenase, ROS, Head and neck cancer

## Abstract

**Background:**

Endocannabinoids have recently drawn attention as promising anti-cancer agents. We previously observed that anandamide (AEA), one of the representative endocannabinoids, effectively inhibited the proliferation of head and neck squamous cell carcinoma (HNSCC) cell lines in a receptor-independent manner. In this study, using HNSCC cell lines, we examined the anti-cancer effects and the mechanisms of action of docosahexaenoyl ethanolamide (DHEA) and N-arachidonoyl-L-alanine (NALA), which are polyunsaturated fatty acid (PUFA)-based ethanolamides like AEA.

**Methods and Results:**

DHEA and NALA were found to effectively inhibit HNSCC cell proliferation. These anti-proliferative effects seemed to be mediated in a cannabinoid receptor-independent manner, since the antagonist of cannabinoid receptor-1 (CB1) and vanilloid receptor-1 (VR1), two endocannabinoid receptors, did not reverse the ability of DHEA and NALA to induce cell death. Instead, we observed an increase in reactive oxygen species (ROS) production and a decrease of phosphorylated Akt as a result of DHEA and NALA treatment. Antioxidants efficiently reversed the inhibition of cell proliferation and the decrease of phosphorylated Akt induced by DHEA and NALA; inhibition of 5-lipoxygenase (5-LO), which is expected to be involved in DHEA- and NALA-degradation pathway, also partially blocked the ability of DHEA and NALA to inhibit cell proliferation and phosphorylated Akt. Interestingly, ROS production as a result of DHEA and NALA treatment was decreased by inhibition of 5-LO.

**Conclusions:**

From these findings, we suggest that ROS production induced by the 5-LO pathway mediates the anti-cancer effects of DHEA and NALA on HNSCC cells. Finally, our findings suggest the possibility of a new cancer-specific therapeutic strategy, which utilizes 5-LO activity rather than inhibiting it.

**Electronic supplementary material:**

The online version of this article (doi:10.1186/s12885-016-2499-3) contains supplementary material, which is available to authorized users.

## Background

Endocannabinoids are endogenously-produced cannabinoids that are involved in a variety of physiological processes (including pain-sensation and memory) through the activation of cannabinoid receptors [1]. Endocannabinoids recently gained attention because cannabis began to be clinically used [2]. More interestingly, these endogenous molecules have been reported to exert cytostatic, apoptotic, and anti-angiogenic effects in different cancer cell lines and cancer xenografts [3–5].

Although the mechanistic actions of endocannabinoids have been revealed in several cancer cell types, the exact mechanisms underlying their anti-cancer action are still unclear. This may be because of the complexity and variety of the signaling pathways that endocannabinoids induce, which seem to involve both receptor-dependent and receptor-independent pathways [6, 7]. Evidence suggests that endocannabinoids might suppress cancer cell viability through the activation of classic cannabinoid receptors such as cannabinoid receptor-1/2 (CB1 and CB2) and vanilloid receptor-1 (VR1). However, increased production of ceramide and reactive oxygen species (ROS), and activation of caspase, PPARs, p38, and JNK signaling are reported to be related to the anti-cancer action of endocannbinoids [8–12]. New putative receptors for endocannabinoids, such as GPR55, have been recently identified, and there is a possibility that these receptors contribute to off-target endocannabinoid effects in order to suppress cancer cell viability [13].

Since cyclooxygenase-2 (COX-2), the enzyme that produces prostanoids from arachidonic acid (AA), is well known to be associated to cell viability in several types of cancer [14], COX-2 has been studied as a useful therapeutic target for the treatment of various cancers [14, 15]. 5-Lipoxygenase (5-LO), the other enzyme involved in AA metabolism, was reported to be overexpressed in some cancer cells [16]. Similar to COX-2, 5-LO is expected to be a promising target for molecular targeted cancer therapy because 5-LO has been identified as being related to carcinogenesis due to its ability to promote cell proliferation and angiogenesis [17–19].

Previously, several groups observed that the cancer cell-killing effects of anandamide (AEA) were mediated through prostamides produced by COX-2 in some types of cancer [20]. These findings are important for molecular targeted cancer therapy, since COX-2 has been found to be highly expressed in many cancer cells. However, we expected that targeting 5-LO, may be another potential therapeutic strategy. In this study, using head and neck squamous cell carcinoma (HNSCC) cancer cells, we investigated the precise role of AA-catabolizing enzymes in regulating the receptor-independent anti-cancer effects of several endocannabinoids that are similar to AA in chemical structure. Since both 5-LO and COX-2 are associated with AA metabolism, we hypothesized that 5-LO might be also be related to the catabolism of some endocannabinoids, including DHEA, EPEA and NALA, all of which are similar in structure to AA. Although we have already analyzed and observed (especially through the induction of angiogenesis) the carcinogenic role of 5-LO in head and neck cancer cells [17], here, we further investigated the possibility of targeting 5-LO as a possible cancer treatment.

## Methods

### Cell culture

SNU-1041, SNU-1066 and SNU-1076 cells (human HNSCC cell lines) were obtained from the Korean Cell Line Bank (Seoul National University, Seoul, Korea), while PCI-1 (human HNSCC cell lines) was obtained from the Pittsburgh Cancer Institute (University of 7Pittsburgh, PA) [17]. HOK 16B is an immortalized cell from pharyngeal mucosa (a gift from Dr. Jeffrey N. Myers in M.D. Anderson Cancer Center, University of Texas) [21]. Cells were maintained at 37 °C in a humidified, 5 % CO_2_, 95 % air atmosphere and routinely sub-cultured using trypsin-EDTA.

### Reagents

Endocannabinoids - docosahexaenoyl ethanolamide (DHEA), eicosapentaenoyl Ethanolamide (EPEA) and N-arachidonoyl-L-alanine (NALA), antagonists of CB1 and VR1 (AM251, cay10448), antioxidants (NAC and GSH), and inhibitors of 5-LO (AA861, zileuton and ebselen) were obtained from Cayman Chemical (Ann Arbor, MI).

### Cell proliferation assay

Cells were seeded in culture plates and incubated for the specific time at 37 °C prior to treatment with specific drugs for the indicated time. After treatment, Cell Counting Kit-8 (Dojindo Lab., Tokyo, Japan) was used to measure cell proliferation according to the manufacturer’s instructions.

### Measurement of apoptosis by Annexin-V staining assay

Apoptosis of SNU-1041 and SNU-1076 by DHEA and NALA was assessed using an Annexin-V staining kit (Koma Biotech, Seoul, Korea). After exposure to 20 μM of DHEA or NALA for 60 h, cells were harvested and washed with cold PBS and re-suspended in binding buffer containing fluorescein isothiocyanate (FITC)-conjugated annexin V protein and propidium iodide. Annexin V binding and PI staining were determined by flow cytometric analysis (Becton Dickinson, San Jose, CA, USA). Apoptotic cells were defined as PI-negative and annexin V-positive.

### Plasmids expressing FAAH and 5-LO

Using each cDNA, we established pcDNA3.1 expressing vectors (pcDNA3.1-lacZ, -FAAH and -5LO). Cells were transfected with 0.5-1 μg of plasmids by electroporation using Microporator MP-100 (NanoEnTek Inc., Seoul, South Korea), following the protocol provided by the manufacturer. Then, cells were seeded in culture plates and incubated for an additional 36 h before another treatment of AEA.

### Transfection of siRNA

Individual siRNAs against COX-2 (D-004557-04), 5-LO (L-004530-00) and non-targeting control (D-001210-01) were obtained from Dharmacon RNA Technologies (Lafayette, CO). The best conditions of siRNAs application (used doses and treatment time) were established beforehand by western blotting and EIA [17]. Cells were transfected with 200 nM of siRNA by electroporation using Microporator MP-100 (NanoEnTek Inc., Seoul, South Korea), following the protocol provided by the manufacturer. Then, cells were seeded in culture plates and incubated for an additional 48 h before another treatment of tested drugs (like DHEA).

### Quantification of PGE_2_ and LTB_4_ production

The amount of the desired factor released by the cells was determined using PGE_2_ or LTB_4_ enzyme immunoassay kits (EIA) (Cayman Chemical, Ann Arbor, MI) according to the manufacturer’s instructions.

### Cell co-culture with transwell system

SNU-1041 cells were transfected with 200 nM of siRNA against 5-LO or non-targeting control and placed at once in the lower side of a transwell (NUNC Company, Denmark) chamber partitioned by a polycarbonate membrane (8.0 μm pore size, Corning Incorporated, Costar). Then SNU-1041 cells (with no transfection) were seeded in the upper side and co-cultured for 48 h. Subsequently, cells were treated with 30 μM of DHEA or NALA for additional 48 h. Both cells (in upper and lower side) were separately applied to the cell proliferation assay (at a total of 96 h).

### Measurements of production of reactive oxygen species (ROS)

The generation of ROS was measured by using the DCFH_2_-DA assay [22]. Intracellular ROS production was determined directly in cell monolayers in black 96-well flat-bottom microtiter plates using a Fluoroskan Ascent FL microplate reader (Labsystems, Sweden). Cells in complete medium were incubated with the indicated drugs for 18 h. To measure the production of ROS, cells were treated with 5 μM DCFH_2_-DA at 37 °C for 30 min, and the fluorescence of DCF was measured at 530 nm after excitation at 485 nm (DCFH_2_-DA, after deacetylation to DCFH_2_, is oxidized intracellularly to its fluorescent derivative DCF). Assays were performed in modified Hank’s buffered salt solution (HBSS).

### Western blot analysis

Denatured protein lysates were resolved by 4–12 % NuPAGE gels (Invitrogen, Carlsbad, CA) and transferred to nitrocellulose membranes (Schleicher & Schuell, Dachen, Germany). The membranes were incubated with anti-5-LO (BD, Franklin Lakes, NJ); anti-p-Akt (Ser473), anti-pan-Akt (Cell signaling, Danvers, MA); or monoclonal anti-β-actin (Santa Cruz Biotechnology, Santa Cruz, CA) for 2 h at room temperature or overnight at 4 °C. Membranes were then washed (4 times) with TBS-T and incubated with horseradish peroxidase-conjugated secondary antibody (Pierce, Rockford, IL) for 1 h. Immunoreactive proteins were visualized by developing them with Lumi-light western blotting substrate (Roche Diagnostics GmbH, Mannheim, Germany), followed by exposure in a LAS-3000 (Fuji Film Co., Tokyo, Japan) according to the manufacturer’s instructions. This was followed by quantitation of specific bands with the Multi Gauge software (Fuji Film Co., Tokyo, Japan).

### Statistical analysis

Data are presented as the mean ± standard deviation (SD) of at least triplicates, or as a representative of 3 separate experiments. Significance was determined between treated and untreated groups by two-sided Student’s *t*-test. *P* values <0.05 were considered statistically significant.

## Results

### DHEA and NALA effectively inhibit the proliferation of HNSCC cell lines

**Fig. 1:**
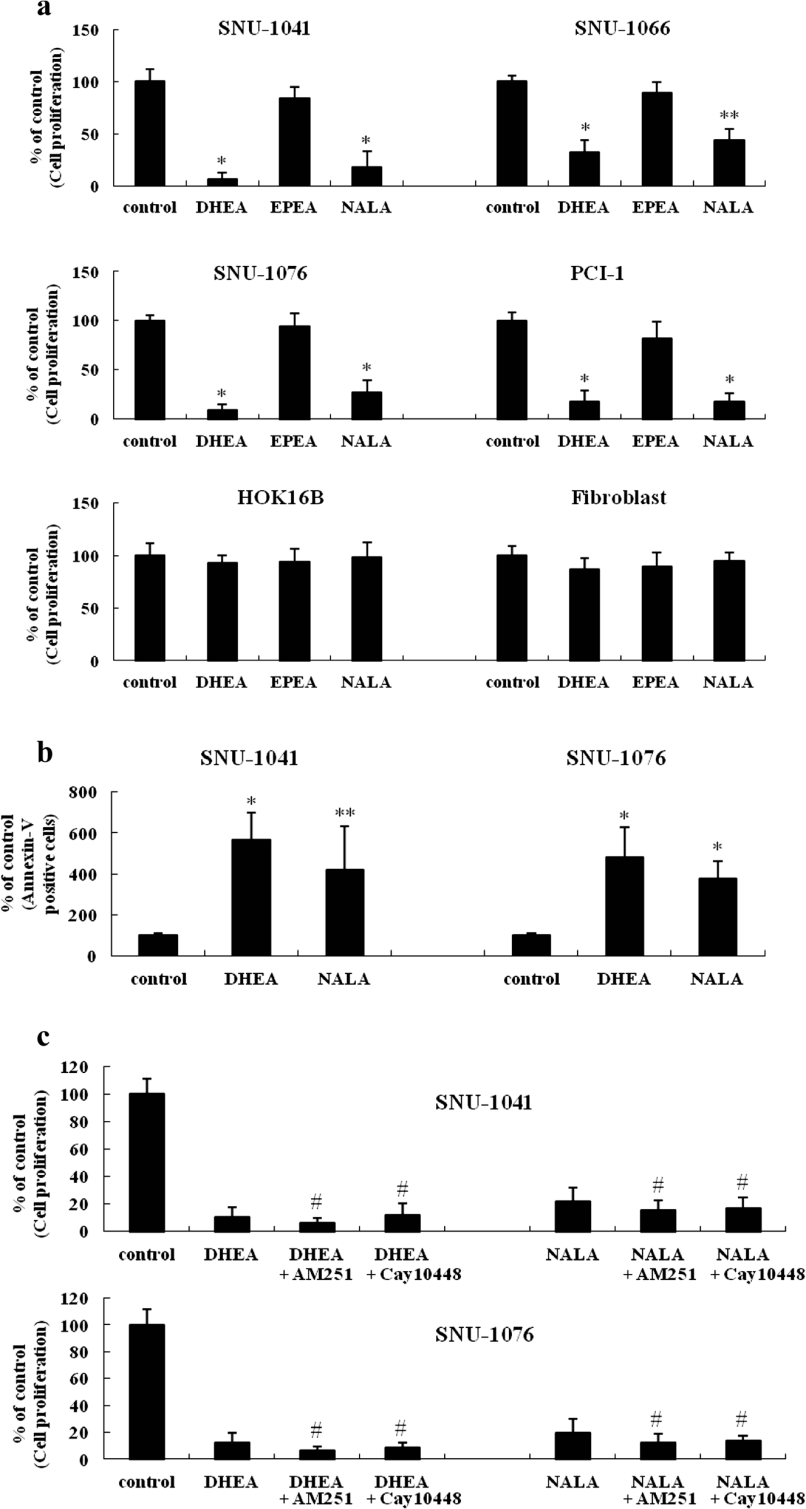
DHEA and NALA effectively inhibit cell proliferation and induce cell death in HNSCC cell lines. **a** Cells were treated with 20 μM of DHEA, EPEA and NALA. At 72 h, cells were subjected to cell proliferation assay. **b** SNU-1041 and SNU-1076 were treated with 20 μM of DHEA and NALA. At 60 h, cells were subjected to Annexin-V staining assay. **c** SNU-1041 and SNU-1076 were treated with DHEA (20 μM) and NALA (20 μM) plus AM251 (2 μM) or cay10448 (2 μM). At 72 h, cells were subjected to cell proliferation assay. Results are expressed as a percentage relative to control (% of control). *P* values were based on comparison with control (*P < 0.001, **P < 0.05) or DHEA/NALA-treated group (^#^ *P* < 0.05)

DHEA and NALA effectively inhibited cell viability in the HNSCC cell lines we tested, but EPEA only had a weak inhibitory effect on cancer cell proliferation (Fig. 1a). Non-cancerous cell lines (HOK16B and fibroblasts) were not affected by DHEA and NALA at the tested doses (10-30 μM) (Fig. 1a). DHEA and NALA effectively induced the cell death in the HNSCC cell lines (Fig. 1b). CB1 is expressed only in SNU-1066 and no expression of CB2 is observed in all the cells tested, while VR1 expression is observed in all cells (in our own study) [23]. We also found that the anti-cancer effect of DHEA and NALA was not reversed by antagonists of the endocannabinoid receptors CB1 and VR1 (AM251 and cay10448) (Fig. 1c). From these observations, we assumed that the anti-cancer effect induced by DHEA and NALA was mediated through a receptor-independent action. The cell lines SNU-1041 and SNU-1076 were chosen for further analysis of the cancer-killing effect of DHEA and NALA.

### The anti-cancer action of DHEA and NALA occurs at an intracellular location

**Fig. 2:**
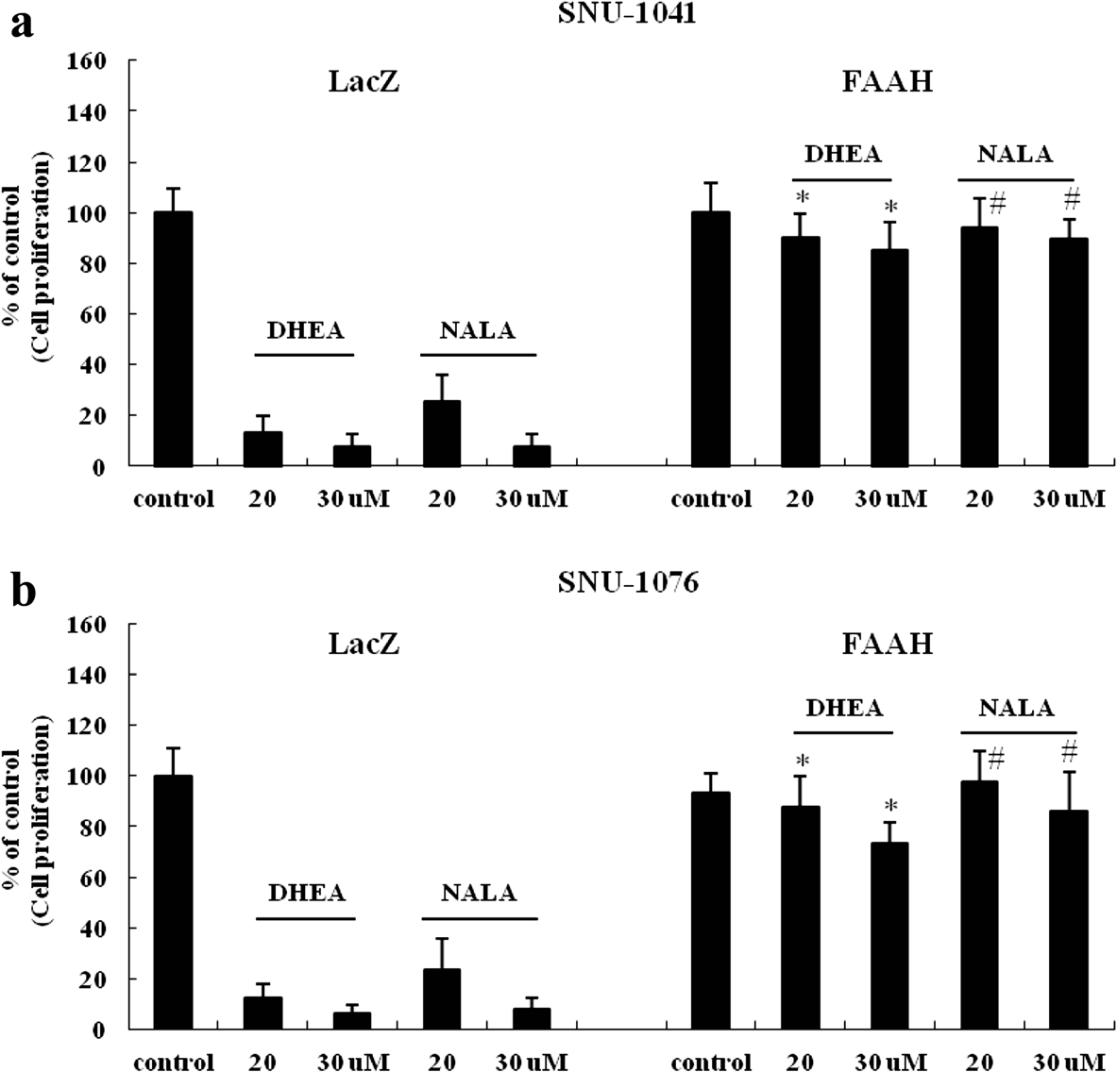
The anti-cancer action of DHEA and NALA occurs at an intracellular location. Plasmids (1 μg) expressing LacZ and FAAH were transfected into (**a**) SNU-1041 and (**b**) SNU-1076 (LacZ expressing plasmid was used for controls). Thirthy-six hours later cells were treated with the indicated concentrations (μM) of DHEA or NALA. At additional 48 h, cells were subjected to cell proliferation assay. Results are expressed as a percentage relative to control (% of control). *P* values are based on a comparison with DHEA-treated group and NALA-treated group in LacZ (*P < 0.001, ^#^ *P* < 0.005)

FAAH is known to catabolize polyunsaturated fatty acid-based endocannabinoids (like AEA) to polyunsaturated fatty acid and ethanolamide [24]. To verify the possibility that DHEA and NALA affected cell viability through a receptor-independent action that occurred after intracellular transport, cells were transfected with plasmids expressing fatty acid amide hydrolase (FAAH). The activity of transfected FAAH was confirmed by using arachidonoyl *p*-nitroaniline-based assay (Additional file 1: Figure S1). We observed that the growth-inhibitory action of DHEA and NALA was completely blocked (Fig. 2). These observations suggested that DHEA and NALA might have anti-cancer effect through intracellular localization by a receptor-independent mechanism in HNSCC cell lines. The used cells in this study had little FAAH activity (data not shown).

### Anti-cancer effect of DHEA and NALA was reversed by inhibition of 5-LO, but not by inhibition of COX-2

**Fig. 3:**
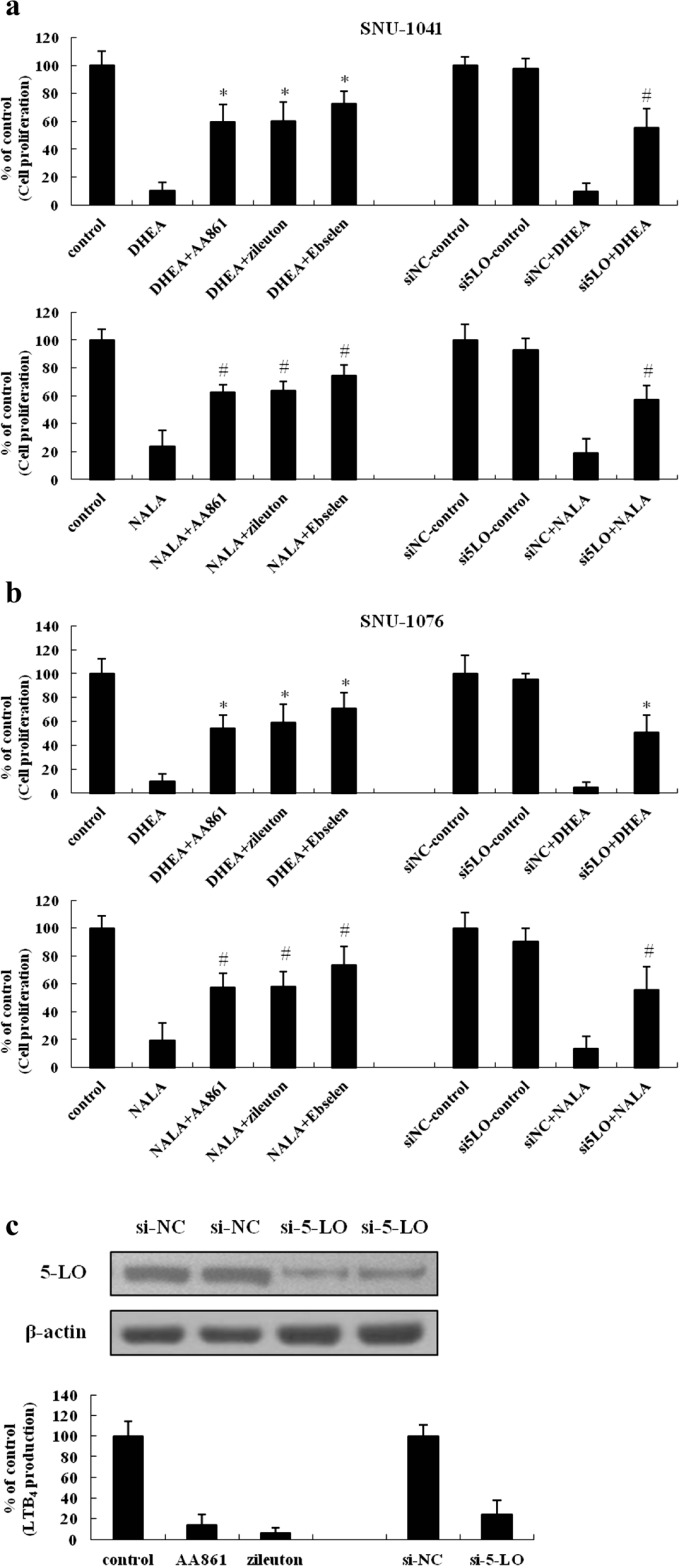
Anti-cancer effect of DHEA and NALA was reversed by inhibition of 5-LO, but not by inhibition of COX-2. (**a**) SNU-1041 and (**b**) SNU-1076 were treated with DHEA or NALA (20 μM) plus AA861 (5 μM) or zileuton (5 μM) or Ebselen (5 μM). At 72 h, cells were subjected to cell proliferation assay (*Left*). The siRNA of 5-LO was transfected at 200 nM doses (the si-NC was used for negative control of siRNA). Forty-eight hours later cells were treated with DHEA or NALA (20 μM). At additional 48 h, cells were subjected to cell proliferation assay (*Right*). Results are expressed as a percentage relative to control (% of control). *P* values were based on comparison with DHEA-treated group and NALA-treated group (*P < 0.005, ^#^ *P* < 0.01). **c** 5-LO siRNA was transfected into SNU-1076 cells. At 48 h, total cell lysates were prepared and the expression of 5-LO was determined by western blotting (*upper*). Data are presented as a representative of 3 separate experiments. At 48 h, cells were treated with 20 μM of arachidonic acid. After an additional 2 h, cultured media were collected and applied to LTB_4_ EIA (*lower*). The inhibitory effect of 5-LO siRNA was compared with that of 5-LO inhibitors – AA861 and zileuton. Results are expressed as a percentage relative to the control (% of control)

AEA, which is structurally similar to AA, has been reported to have an anti-cancer effect when it is catabolized by COX-2 [20]. Therefore, we hypothesized that the mechanism by which DHEA and NALA inhibited cell proliferation might also be a result of their catabolism by COX-2. However, we found that inhibition of COX-2 had no effect on the ability of DHEA and NALA to inhibit cell proliferation of HNSCC (Additional file 2: Figure S2). Next, we tried to investigate if 5-LO might regulate the ability of DHEA and NALA to inhibit cell proliferation. The high expression and activity of 5-LO in HNSCC cells were already measured in our previous study [17]. Cells were treated with 5-LO inhibitors (AA861, zileuton, and ebselen) and 5-LO siRNAs together with DHEA or NALA before cell proliferation was measured. We were able to demonstrate that 5-LO mediated the growth-inhibitory actions of DHEA and NALA in SNU-1041 (Fig. 3a) as well as in SNU-1076 (Fig. 3b). The inhibition of 5-LO activity by its inhibitors and by its siRNA was confirmed by using an leukotriene B_4_ (LTB_4_) EIA (Fig. 3c).

### The anti-cancer effects of DHEA and NALA are not mediated by any products generated by the 5-LO pathway

**Fig. 4:**
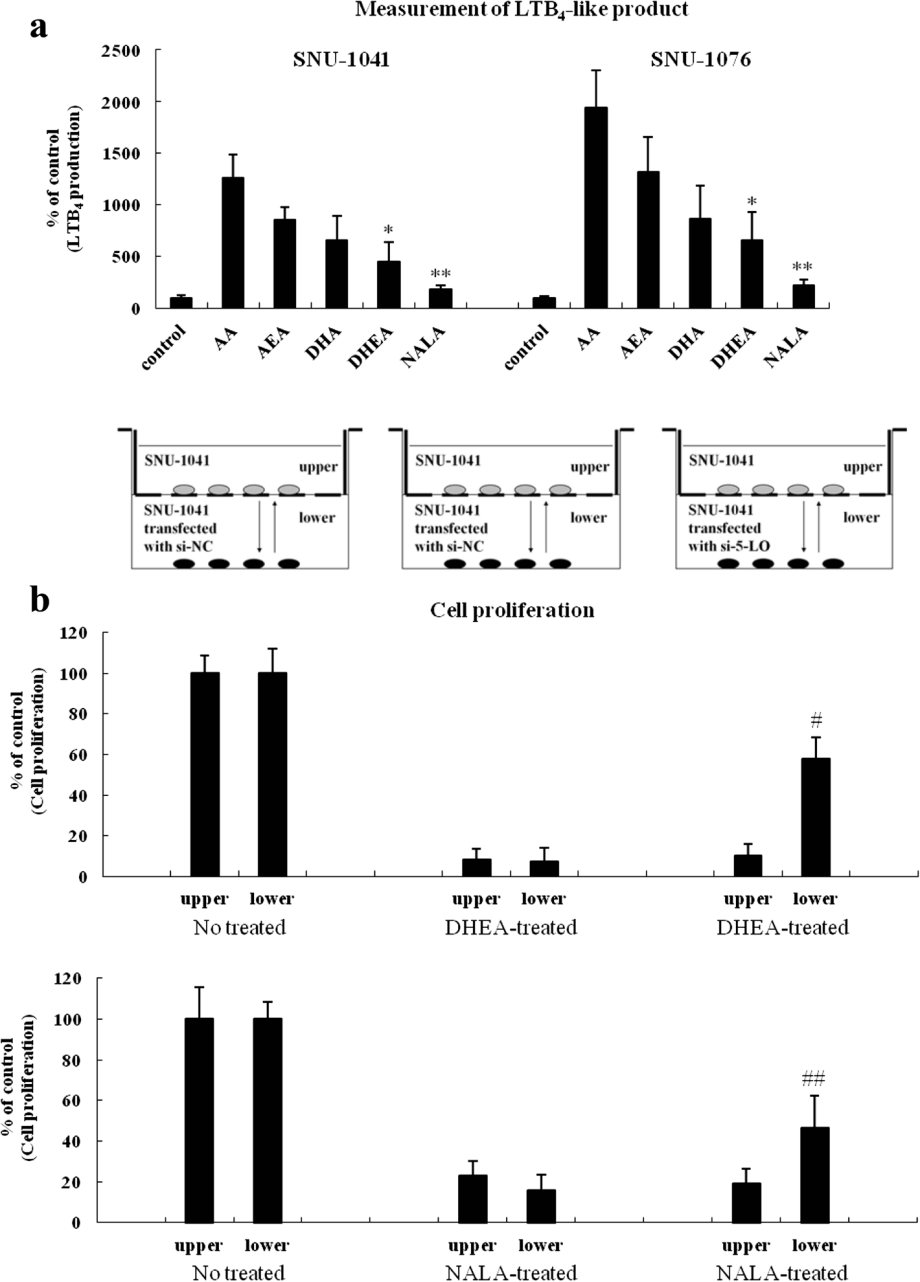
The anti-cancer effects of DHEA and NALA are not mediated by any products generated by the 5-LO pathway. **a** SNU-1041 and SNU-1076 were treated with AA, AEA, DHA, DHEA and NALA (20 μM). At 4 h, cells were subjected to the LTB_4_ EIA. Results are expressed as a percentage relative to control (% of control). **b** SNU-1041 cells were transfected with 200 nM of siRNA against 5-LO or si-NC and placed at once in the lower side of a transwell chamber. Then SNU-1041 cells (with no transfection) were seeded in the upper part and co-cultured for 48 h. Subsequently, cells were treated with 30 μM of DHEA or NALA for additional 48 h. Both cells (*in upper* and *lower side*) were separately applied to the cell proliferation assay. Results are expressed as a percentage relative to control (% of control). *P* values were based on comparison with control (*P < 0.01, **P < 0.05) or DHEA/NALA-treated group (^#^ *P* < 0.005, ^##^ *P* < 0.05)

Because of the structural similarity between AA and DHEA/NALA, we could detect weak LTB_4_-like products synthesized by 5-LO from DHEA and NALA using an LTB_4_ EIA kit (Fig. 4a). However, when cells transfected with siRNAs of negative control (NC) or 5-LO were co-cultured with cells in upper side (with basic condition) and treated with DHEA and NALA, we observed that cell viability was partially reversed only in 5-LO siRNA-transfected cells (Fig. 4b).

### DHEA and NALA increase ROS production

**Fig. 5:**
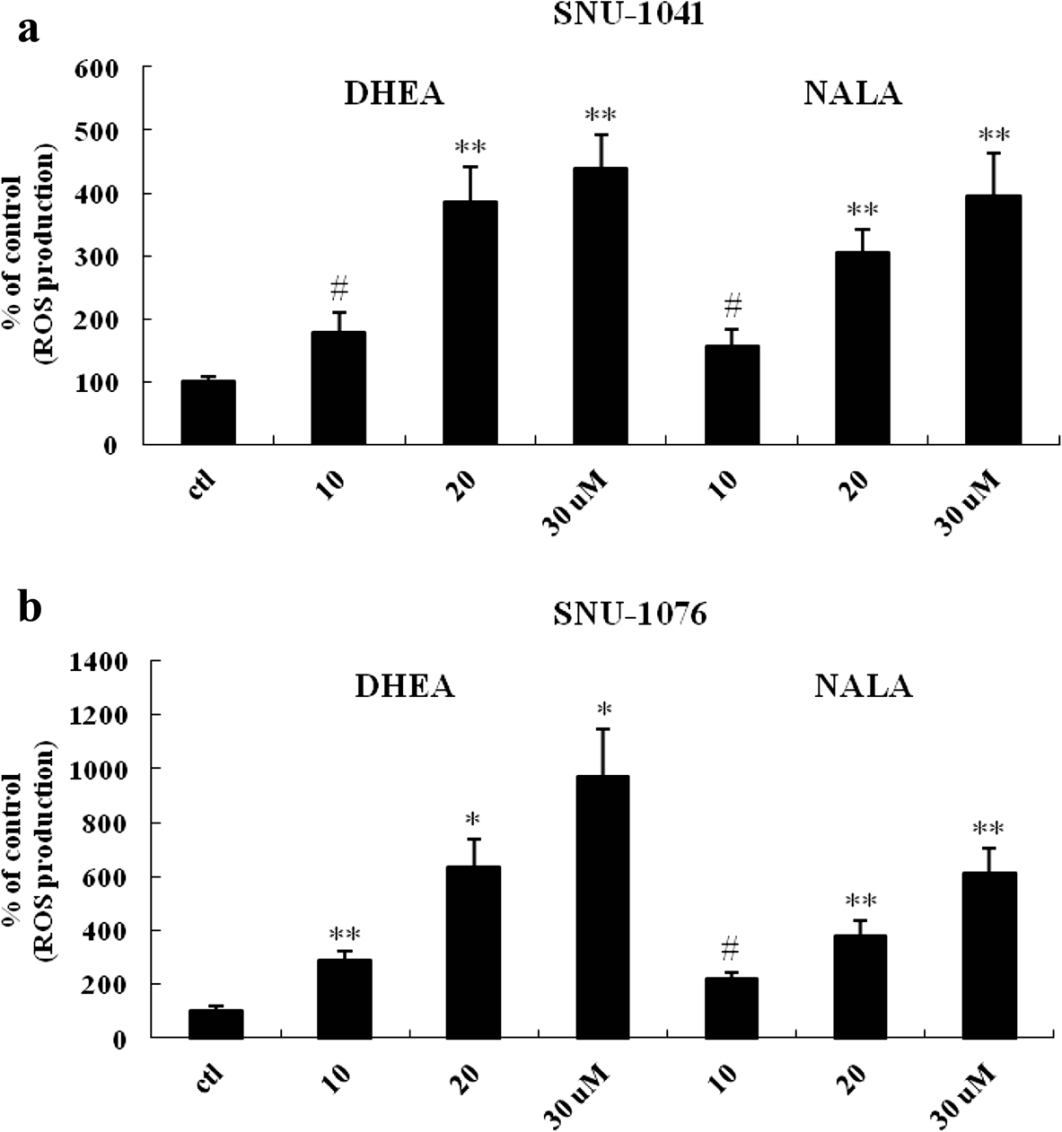
DHEA and NALA increase ROS production. **a** SNU-1041 and (**b**) SNU-1076 were treated with the indicated concentrations (μM) of DHEA and NALA. At 18 h, cells were subjected to the DCFH_2_-DA assay to measure the change of ROS level. Results are expressed as a percentage relative to control (% of control). *P* values were based on comparison with control (*P < 0.001, **P < 0.005, ^#^ *P* < 0.01)

In our own study, we observed that AEA increased intracellular oxidative stress, including lipid peroxidation [23]. Since DHEA and NALA are very similar to AEA, we assumed that DHEA and NALA might affect cell viability by increasing intracellular ROS production. As expected, we observed an increase in ROS production as a result of DHEA and NALA treatment in SNU-1041 (Fig. 5a) and SNU-1076 (Fig. 5b). These data suggest that ROS production induced by DHEA and NALA seems to be involved in mediating the anti-cancer effects of DHEA and NALA in HNSCC cells.

### 5-LO inhibition as well as antioxidant treatment partially reversed DHEA- and NALA-inhibited cell proliferation

**Fig. 6:**
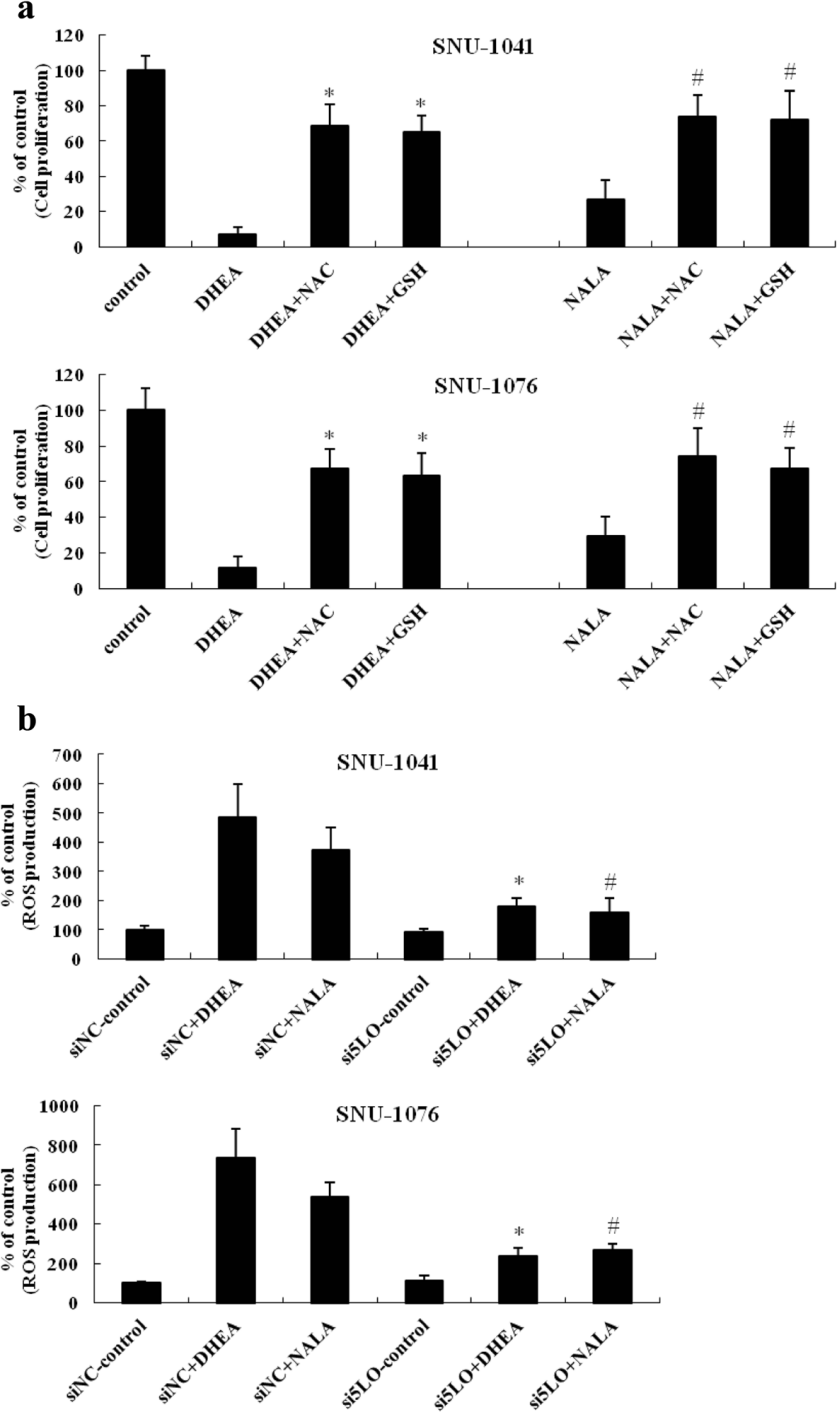
5-LO inhibition as well as antioxidant treatment partially reversed DHEA- and NALA-inhibited cell proliferation. **a** Cells were treated with 20 μM of DHEA and NALA plus NAC (1 mM) or GSH (2 mM). At 72 h, cells were subjected to cell proliferation assay. Results are expressed as a percentage relative to control (% of control). *P* values were based on comparison with DHEA-treated group and NALA-treated group (*P < 0.001, ^#^ *P* < 0.01). **b** Cells were transfected at 200 nM doses of 5-LO siRNA (the siNC was used for negative control of siRNA). Forty-eight hours later cells were treated with DHEA or NALA (20 μM). At additional 18 h, cells were subjected to the DCFH_2_-DA assay to measure the change of ROS level. Results are expressed as a percentage relative to control (% of control). *P* values were based on comparison with DHEA-treated group and NALA-treated group in siNC (*P < 0.01, ^#^ *P* < 0.05)

Next, to identify the role of increased ROS in the ability of DHEA and NALA to inhibit cell proliferation, we treated SNU-1041 with DHEA/NALA and the antioxidants NAC and GSH. The antioxidants partially reversed DHEA-/NALA-inhibited cell proliferation (Fig. 6a). Together with Fig. 5, this finding confirms that DHEA-/NALA-induced ROS might play a role in the anti-cancer effect of DHEA and NALA on HNSCC cells. In addition, we found that 5-LO siRNAs blocked the increase of DHEA/NALA-induced ROS production in SNU-1041 and SNU-1076 (Fig. 6b).

### 5-LO-induced ROS mediates the decrease of phosphorylated Akt by DHEA and NALA

**Fig. 7:**
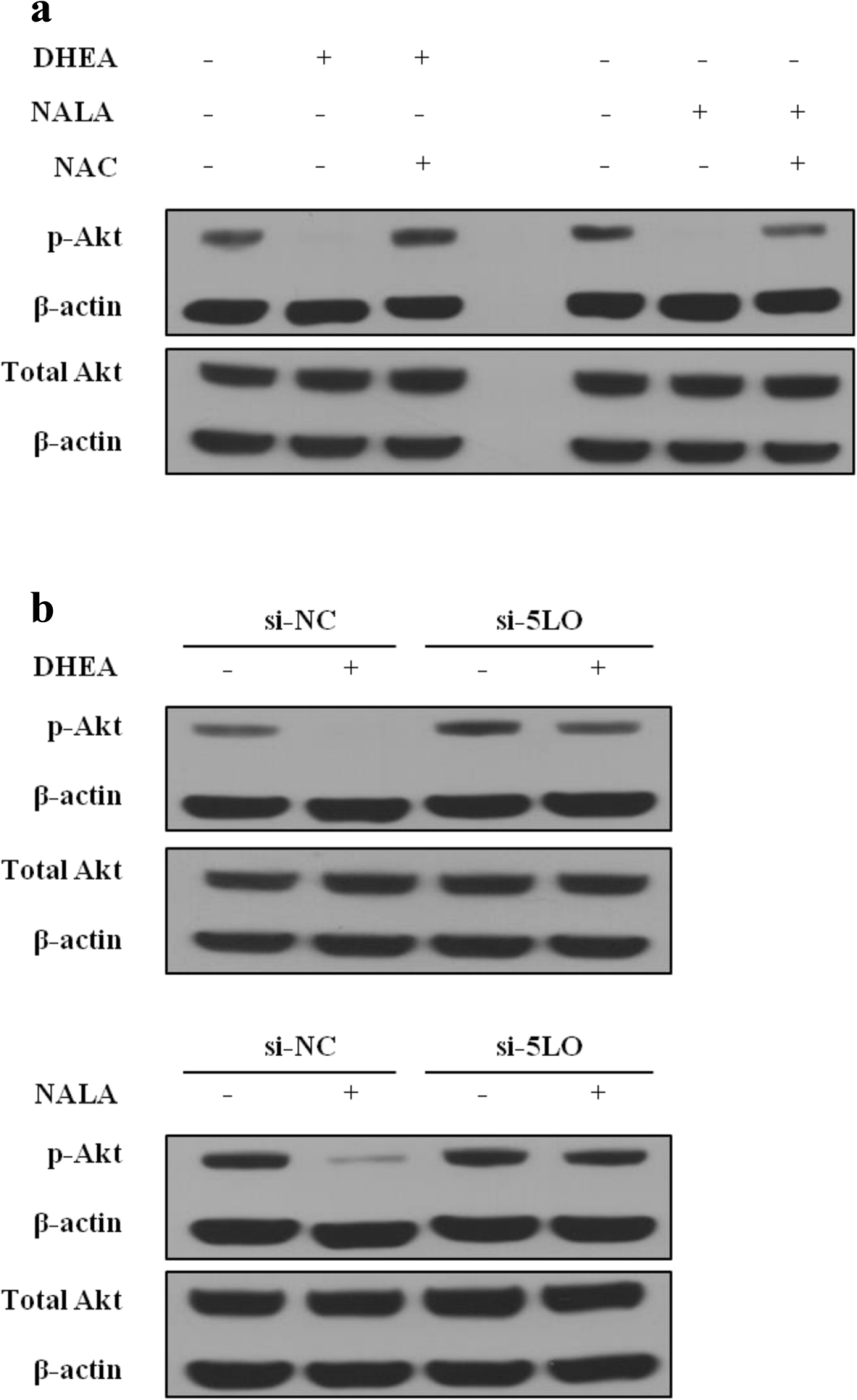
5-LO-induced ROS mediates the decrease of Akt activity by DHEA and NALA. **a** SNU-1041 cells were treated with DHEA or NALA (40 μM) plus NAC (1 mM). At 24 h, cells were harvested and applied to western blotting. **b** Cells were transfected with the indicated siRNA (200 nM). Forty-eight hours later cells were treated with DHEA or NALA (40 μM). After additional 24 h, cells were harvested and applied to western blotting (si-NC was used for control). The membranes were incubated with anti-β-actin plus anti-p-Akt or anti-pan-Akt for 2 h at room temperature (β-actin was used to show an housekeeping gene). Data are presented as a representative of 3 separate experiments

It was already known that Akt activity is important in maintaining the cell viability of several cancer cells, including HNSCC cells [25, 26]. To identify the role of increased ROS in the ability of DHEA/NALA to affect the phosphorylated form of Akt in HNSCC cells, we treated SNU-1041 with DHEA/NALA and the antioxidants NAC. DHEA and NALA decreased the phosphorylated form of Akt and the antioxidants reversed DHEA/NALA-inhibited p-Akt in SNU-1041 (Fig. 7a). In addition, we found that 5-LO inhibition by siRNAs reversed the decrease of DHEA/NALA-inhibited p-Akt in SNU-1041 (Fig. 7b).

### Exogenous transfection of plasmids expressing 5-LO promotes the anti-cancer action of DHEA and NALA in HNSCC cells

**Fig. 8:**
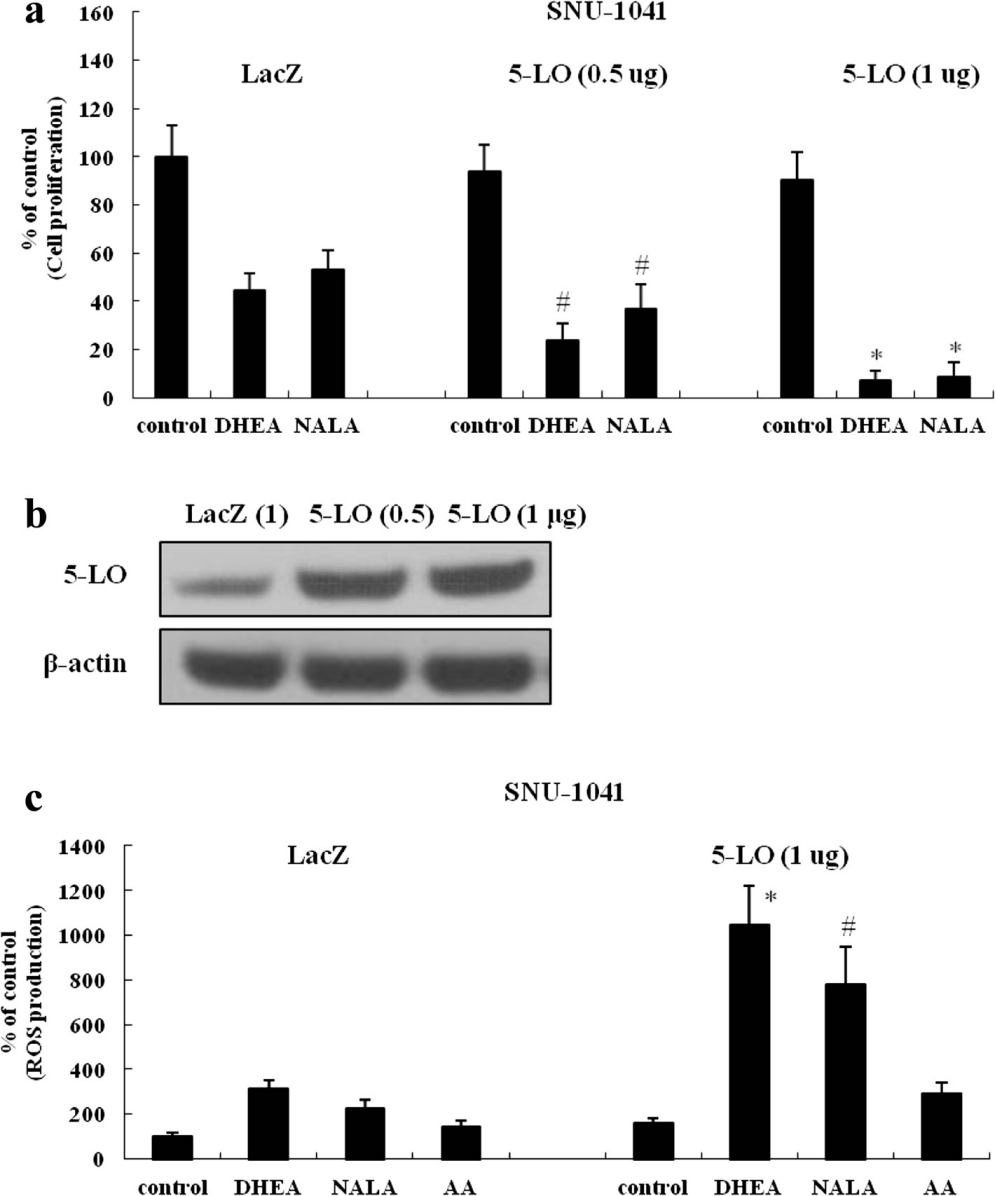
Exogenous transfection of plasmids expressing 5-LO promotes the anti-cancer action of DHEA and NALA in HNSCC cells. **a** Plasmids (0.5-1 μg) expressing 5-LO were transfected into SNU-1041 (LacZ expressing plasmid was used for controls). Thirty-six hours later cells were treated with DHEA and NALA (10 μM). At additional 48 h, cells were subjected to cell proliferation assay. Results are expressed as a percentage relative to control (% of control). *P* values were based on comparison with DHEA-treated group and NALA-treated group in LacZ (*P < 0.01, ^#^ *P* < 0.05). **b** Plasmids (0.5-1 μg) expressing LacZ and 5-LO were transfected into SNU-1041 cells. At 36 h, total cell lysates were prepared, and the expression of 5-LO was determined by western blotting. Data are presented as a representative of 3 separate experiments. **c** Plasmids (1 μg) expressing 5-LO were transfected into SNU-1041. Thirty-six hours later cells were treated with DHEA, NALA and AA (10 μM). At additional 18 h, cells were subjected to the DCFH_2_-DA assay to measure the change of ROS level. Results are expressed as a percentage relative to control (% of control). *P* values were based on comparison with DHEA-treated group and NALA-treated group in LacZ (*P < 0.005, ^#^ *P* < 0.01)

Finally, we investigated the effect of enhanced 5-LO activity on the anti-cancer action of DHEA and NALA in SNU-1041. Transfecting cells with plasmids expressing 5-LO, we observed that the growth-inhibitory activity of DHEA and NALA significantly improved with increasing 5-LO expression (Fig. 8a). The expression of transfected 5-LO was verified by western blotting (Fig. 8b). In addition, ROS production in the presence of DHEA or NALA increased proportionally with expression of 5-LO, which was more prominently than in the presence of AA (the basic substrate of 5-LO pathway) (Fig. 8c).

## Discussion

Since psychotropic side effects by cannabis are reported to be mediated by classic cannabinoid receptors [1], there might be some concern about the idea of adopting endocannabinoids as a cancer treatment. However, it has been also reported that the cell-killing effect of several endocannabinoids is mediated by cannabinoid receptor-independent mechanisms [6, 7, 23]. In addition to classic receptors like CB1 and CB2, GPR55 and GPR35 were recently reported as putative receptors of endocannabinoids [13, 27]. Given these observations, it might be possible to find a way to avoid the psychotropic side effects of endocannabinoids and use them as chemotherapeutic agents. In our study, we hoped to find a CB receptor-independent effect of the endocannabinoids in order to develop them as new cancer therapeutics without psychotropic side effects.

Although DHEA was reported to activate classic cannabinoid receptors [6], the anti-cancer action of DHEA seemed to be mediated by receptor-independent pathways in our study, since antagonists of cannabinoid receptors had no effect on it. Our observation of the perfect reversal of the anti-cancer effect of DHEA and NALA by transfecting FAAH into HNSCC cells confirms that DHEA and NALA can be degraded by FAAH.

The fact that COX-2 and 5-LO are highly expressed in cancer cells than in non-cancerous cells suggests that they might be useful molecular targets for cancer therapy [18, 28]. Their inhibition has been shown to have efficient suppressive effects on cancer cell viability in several types of cancer, such as colon cancer [14, 19]. In our previous study using HNSCC cells, we observed little anti-proliferative effect by inhibiting COX-2 and 5-LO directly [29]. However, in this study, we observed that COX-2 and/or 5-LO activity might be able to promote the cell-killing action induced by some endocannabinoids. This observation suggests that COX-2 and/or 5-LO might be used as specific targets for cancer therapy in ways other than simply inhibiting their activities. Indeed, we identified that DHEA and NALA were able to kill HNSCC cells through 5-LO-mediated ROS production in a receptor-independent manner, even though HNSCC cells might have expression of their receptors such as CB1 and/or VR1.

Until now, it has not been reported that endocannabinoids like DHEA and NALA might be the substrates for 5-LO, even though various polyunsaturated fatty acid (PUFAs) like DHA are known to be degraded by 5-LO [30]. We could efficiently detect LTB_4_-like products generated from DHA and AEA by 5-LO, but could only detect low levels of the products from DHEA and NALA (Fig. 4a). Since SNU-1041 and SNU-1076 have little FAAH activity, we assumed that we could detect LTB_4_-like products directly generated from DHEA and NALA, not those from DHA and AA converted by FAAH.

In cell co-culture experiment, we observed that inhibition of cell viability by DHEA and NALA treatment was partially reversed in 5-LO siRNA-transfected cells of lower side (Fig. 4c and d). It means that the anti-cancer effects of DHEA and NALA are not mediated by LTB_4_-like products generated by the 5-LO pathway but mediated by other mechanisms such as ROS production, which should be induced through the processes of oxygenation and peroxidation by 5-LO. If any end-products of 5-LO released to culture medium showed cell killing action, 5-LO siRNA-transfected cells in lower chamber should have been killed as well.

Other studies also observed the increase of intracellular oxidative stress during AA metabolism, independently of produced eicosanoids [31, 32]. Furthermore, 5-LO activating protein (FLAP) and leukotriene C4 (LTC_4_) synthase are included in the membrane associated proteins in the eicosanoid and glutathione metabolism (MAPEG) superfamily related with glutathione-dependent catalysis [33]. FLAP and LTC_4_ synthase might cause glutathione depletion (which leads to increased ROS) in the conversion of AA to leukotrienes by 5-LO [34].

**Fig. 9:**
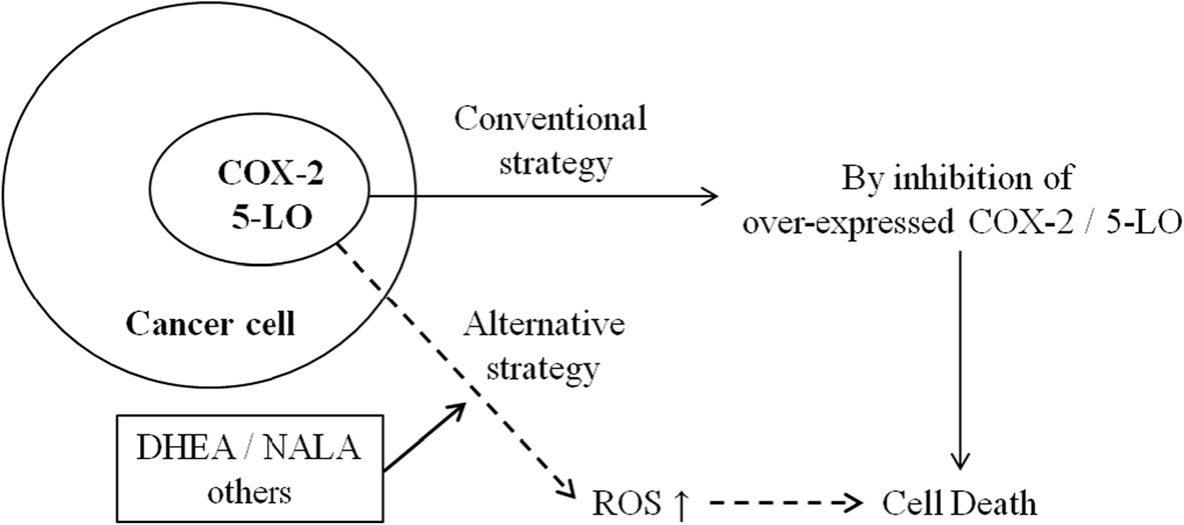
The possibility of development of a new alternative strategy that can utilize COX-2 or 5-LO activity itself in cancer cells. Considering our observation that DHEA and NALA produced more ROS through the 5-LO pathway than AA, we suggest the possibility of development of a new alternative strategy that can utilize COX-2 or 5-LO activity itself in cancer cells but is different from direct inhibition of COX-2 or 5-LO

In particular, the observation that DHEA and NALA produced more ROS through the 5-LO pathway than AA is very interesting and suggests the possibility of development of a new alternative strategy that is different from direct inhibition of COX-2 or 5-LO (Fig. 9). By properly exploiting the characteristics of DHEA and NALA, we may be able to develop novel analogs of endocannabinoids with an ability to efficiently induce 5-LO-mediated ROS production without activating cannabinoid receptors which induce psychotropic side effects. Even though COX-2 was found not to mediate ROS production by DHEA and NALA in this study, more research is needed to elucidate any relationship between several endocannabinoids and COX-2 as well as 5-LO in various cancer cells.

Akt pathway is important in maintaining the cell viability of several cancer cells, including HNSCC cells [25, 26]. Even though it was reported that ROS might affect Akt pathway positively or negatively, depending on tested cell type [35], we observed the decrease of phosphorylated form of Akt by ROS in our cell model. Since inhibition of p-Akt is likely to lead to the decreased cell viability, we think that cell death of HNSCC cells by DHEA and NALA was mediated at least partially through Akt inhibition by increased ROS production.

The fact that inhibition of 5-LO did not completely reverse the effects of DHEA and NALA suggests that the 5-LO pathway is not the only pathway involved in the DHEA- and NALA-mediated inhibition of HNSCC proliferation and Akt phosphorylation. Considering that the identification of new endocannabinoid receptors is still under investigation, we think that further study is needed to investigate the possibility of the involvement of unknown receptors and other action mechanisms (not off-target effects) in the anticancer effects of some endocannabinoids such as DHEA and NALA in order to enhance their utility as new anticancer agents. In addition, although we suggest the possibility of using the 5-LO activity of cancer cells to kill these cells, further study is needed to investigate the differing roles of the 5-LO pathway in the homeostasis of various types of cancer cells because 5-LO inhibition may provide a novel therapeutic strategy for some cancers such as prostate cancer [36–38].

Here, for the first time, we showed that 5-LO might be related to the catabolism of some endocannabinoids, including DHEA and NALA, and observed that 5-LO could mediate the cell-killing action of DHEA and NALA by up-regulating ROS production in HNSCC cells. Since it was identified that DHEA could be physiologically synthesized from DHA in the human body and NALA from AA, the application of proper doses of DHEA and NALA would be clinically effective, non-toxic anti-cancer treatments. Our observations suggest the possibility that DHEA, induced by the dietary supplement DHA, might mediate an anti-cancer effect in some cancers such as HNSCC.

## Conclusions

From these findings, we suggest that ROS production induced by the 5-LO pathway mediates the anti-cancer effects of DHEA and NALA on HNSCC cells. Finally, our findings suggest the possibility of a new cancer-specific therapeutic strategy, which utilizes 5-LO activity rather than inhibiting it.

## Abbreviations

5-LO, 5-lipoxygenase; AEA (Anandamide), Arachidonoyl ethanolamide; CB1, cannabinoid receptor-1; COX-2, cyclooxygenase-2; DHEA, docosahexaenoyl ethanolamide; FAAH, fatty acid amide hydrolase; HNSCC, head and neck squamous cell carcinoma; LTB_4_, Leukotriene B_4_; NALA, N-arachidonoyl-L-alanine; PGE_2_, prostaglandin E_2_; PUFA, polyunsaturated fatty acid; ROS, Reactive oxygen species; siRNA, small interfering RNA; VR1, vanilloid receptor-1

## Additional files

Additional file 1: Figure S1. Activity of FAAH in trasfected SNU-1041 cells. (PPTX 73 kb) Additional file 2: Figure S2. Anti-cancer effect of DHEA and NALA was not reversed by inhibition of COX-2. (PPTX 75 kb)
