# Supplementary material for: 5-lipoxygenase mediates docosahexaenoyl ethanolamide and N-arachidonoyl-L-alanine-induced reactive oxygen species production and inhibition of proliferation of head and neck squamous cell carcinoma cells
Source: BMC Cancer. 2016 Jul 13;16:458. doi: 10.1186/s12885-016-2499-3 (PMC4942960; doi:10.1186/s12885-016-2499-3)
Supplement: Additional file 1: Figure S1. — Activity of FAAH in trasfected SNU-1041 cells. (PPTX 73 kb) [file 12885_2016_2499_MOESM1_ESM.pptx]

## Slide 1
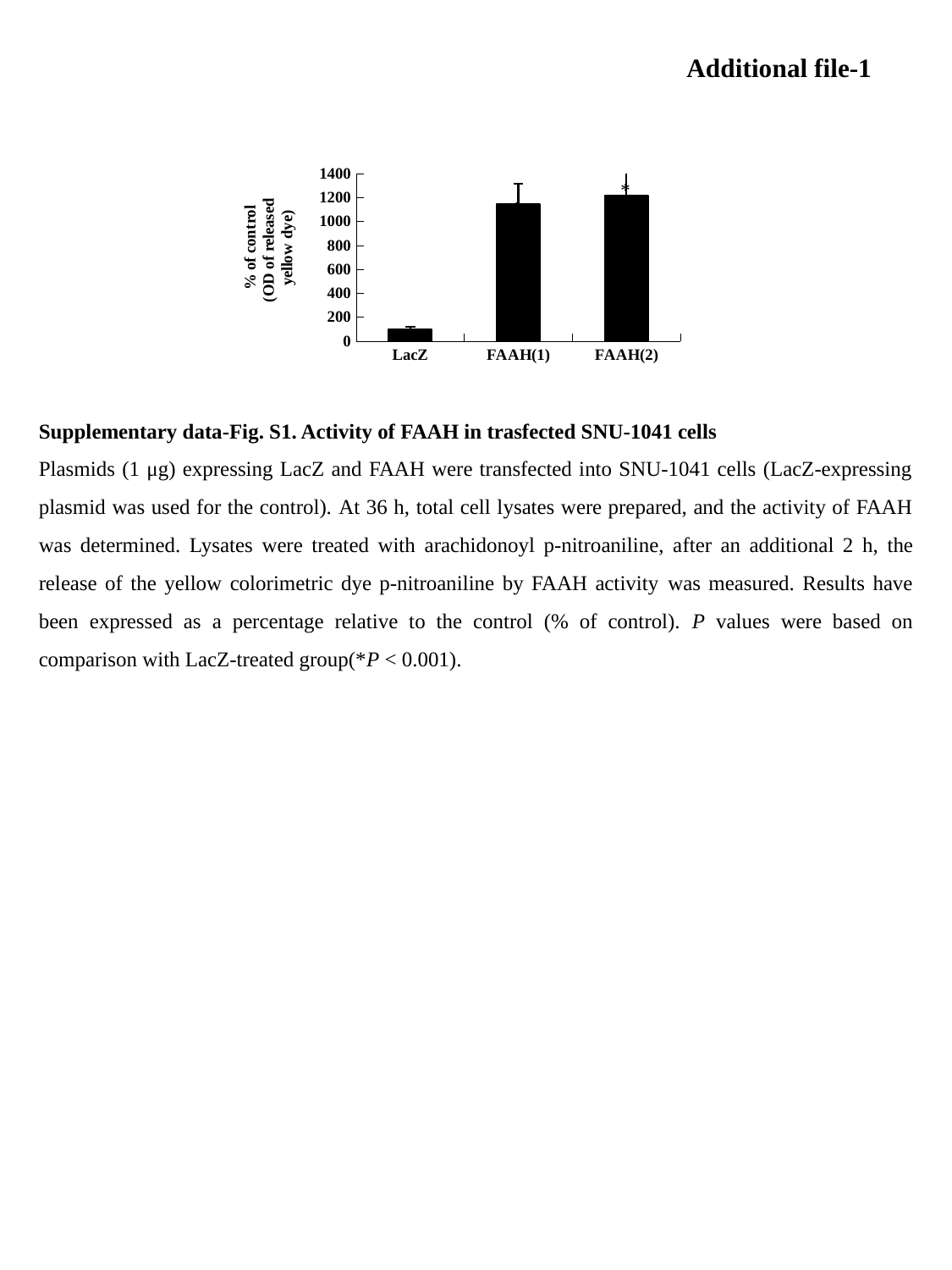

Additional file-1
### Chart
| Category | % of control |
|---|---|
| LacZ | 100.0 |
| FAAH(1) | 1143.5 |
| FAAH(2) | 1219.1 |Supplementary data-Fig. S1. Activity of FAAH in trasfected SNU-1041 cells
Plasmids (1 μg) expressing LacZ and FAAH were transfected into SNU-1041 cells (LacZ-expressing plasmid was used for the control). At 36 h, total cell lysates were prepared, and the activity of FAAH was determined. Lysates were treated with arachidonoyl p-nitroaniline, after an additional 2 h, the release of the yellow colorimetric dye p-nitroaniline by FAAH activity was measured. Results have been expressed as a percentage relative to the control (% of control). P values were based on comparison with LacZ-treated group(*P < 0.001).
*
*
