# Supplementary material for: 5-lipoxygenase mediates docosahexaenoyl ethanolamide and N-arachidonoyl-L-alanine-induced reactive oxygen species production and inhibition of proliferation of head and neck squamous cell carcinoma cells
Source: BMC Cancer. 2016 Jul 13;16:458. doi: 10.1186/s12885-016-2499-3 (PMC4942960; doi:10.1186/s12885-016-2499-3)
Supplement: Additional file 2: Figure S2. — Anti-cancer effect of DHEA and NALA was not reversed by inhibition of COX-2. (PPTX 75 kb) [file 12885_2016_2499_MOESM2_ESM.pptx]

## Slide 1
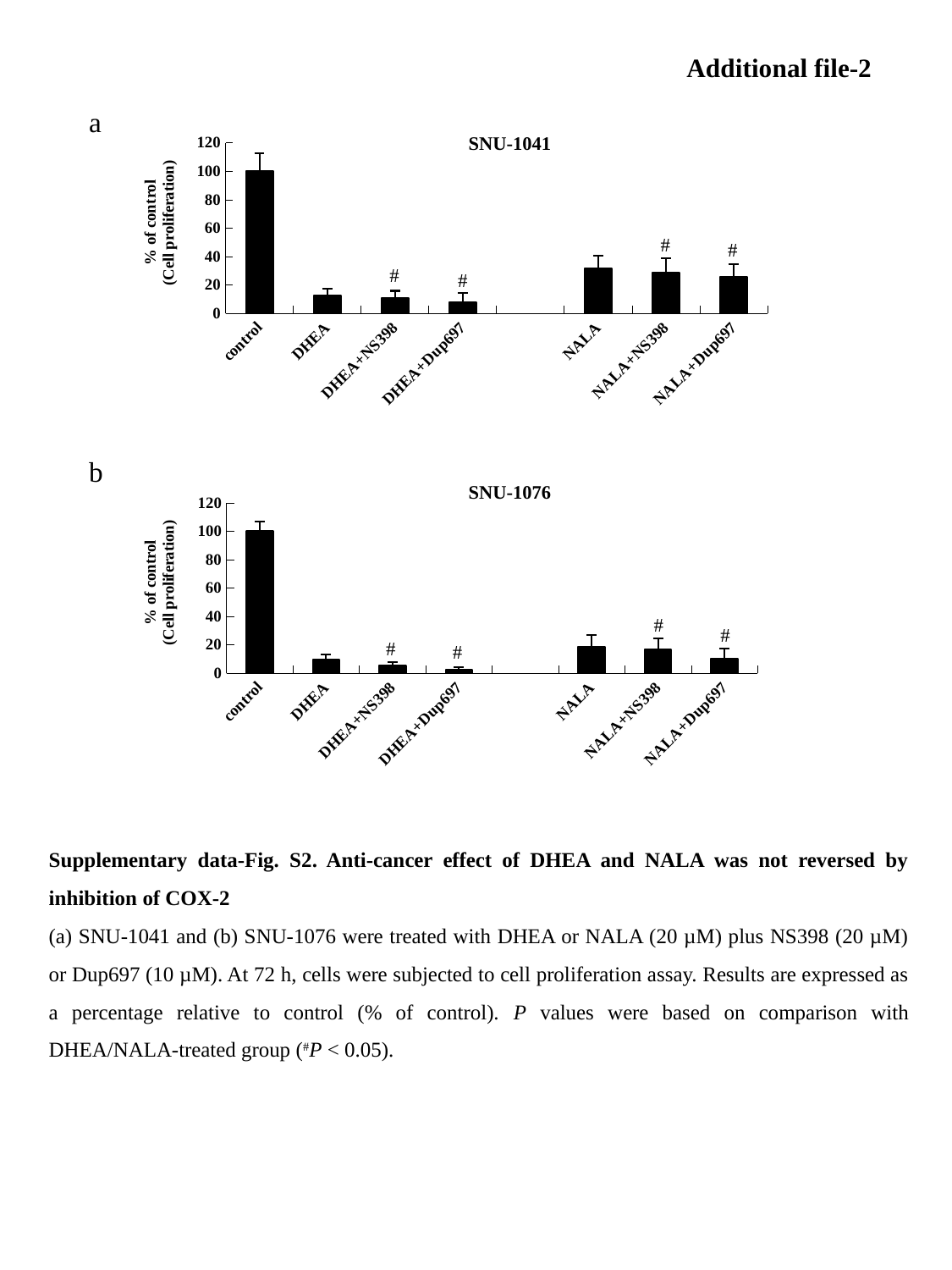

Additional file-2
a
### Chart
| Category | % of control |
|---|---|
| control | 100.0 |
| DHEA | 12.7 |
| DHEA+NS398 | 10.5 |
| DHEA+Dup697 | 7.6 |
| | None |
| NALA | 31.5 |
| NALA+NS398 | 28.4 |
| NALA+Dup697 | 25.5 |SNU-1041
#
#
#
#
b
SNU-1076
### Chart
| Category | % of control |
|---|---|
| control | 100.0 |
| DHEA | 9.5 |
| DHEA+NS398 | 5.6 |
| DHEA+Dup697 | 2.3 |
| | None |
| NALA | 18.4 |
| NALA+NS398 | 16.9 |
| NALA+Dup697 | 10.5 |#
#
#
#
Supplementary data-Fig. S2. Anti-cancer effect of DHEA and NALA was not reversed by inhibition of COX-2
(a) SNU-1041 and (b) SNU-1076 were treated with DHEA or NALA (20 µM) plus NS398 (20 µM) or Dup697 (10 µM). At 72 h, cells were subjected to cell proliferation assay. Results are expressed as a percentage relative to control (% of control). P values were based on comparison with DHEA/NALA-treated group (#P < 0.05).
